# Shifting to Value-Based Principles in Sickness Insurance: Challenges in Changing Roles and Culture

**DOI:** 10.1007/s10926-018-9759-9

**Published:** 2018-02-12

**Authors:** Christian Ståhl, Frieda Andersson

**Affiliations:** 1 Division of Community Medicine, Department of Medical and Health Services, Linköping University, 581 83 Linköping, Sweden; 2 HELIX Competence Centre, Linköping University, Linköping, Sweden; 3 Division of Nursing Science, Department of Medical and Health Sciences, Linköping University, 581 83 Linköping, Sweden

**Keywords:** New Public Management, Lean, Social insurance, Work disability, Accountability, Sweden

## Abstract

*Purpose* Management principles in insurance agencies influence how benefits are administered, and how return to work processes for clients are managed and supported. This study analyses a change in managerial principles within the Swedish Sickness Insurance Agency, and how this has influenced the role of insurance officials in relation to discretion and accountability, and their relationship to clients. *Methods* The study is based on a qualitative approach comprising 57 interviews with officials and managers in four insurance offices. *Results* The reforms have led to a change in how public and professional accountability is defined, where the focus is shifted from routines and performance measurements toward professional discretion and the quality of encounters. However, the results show how these changes are interpreted differently across different layers of the organization, where New Public Management principles prevail in how line managers give feedback on and reward the work of officials. *Conclusions* The study illustrates how the introduction of new principles to promote officials’ discretion does not easily bypass longstanding management strategies, in this case managing accountability through top-down performance measures. The study points out the importance for public organizations to reconcile new organizational principles with the current organizational culture and how this is manifested through managerial styles, which may be resistant to change. Promoting client-oriented and value-driven approaches in client work hence needs to acknowledge the importance of organizational culture, and to secure that changes are reflected in organizational procedures and routines.

## Introduction

New Public Management (NPM) has been a dominant principle for public agencies for several decades, where the principles have their background in a strive to design more efficient government services by adopting principles from private corporations: central aspects are to treat recipients as customers rather than citizens, and to emphasize constant monitoring of service performance to secure efficient use of resources [1]. Over the last decade or so, there has been a development away from the market-oriented principles of NPM into more value-driven principles of operation, e.g., through emphasizing democratic values, public interests and quality of services, which also have effects on the roles of the officials working within the organizations [1, 2]. One example of a more value-based principle is lean, which is both a rhetoric and a set of managerial tools; key elements are to increase customer value, reducing ‘waste’ in the organizational processes, and increasing quality through including employees in improvement work [2, 3]. The emerging value-based approach to public administration has its background in a networked and complex environment including several actors, where government agencies find themselves acting not only as administrators, but also as catalysts, collaborators or conveners [1]. In a sickness and return to work (RTW) setting, this is of importance given the multi-stakeholder environment in which administration of work disability and RTW promotion is taking place [4, 5]. Changes in objectives of public organizations also influence the approach to accountability and key values within those organizations, calling for a focus on dialogue and deliberation with multiple actors, and to move from control-oriented to trust-oriented management [6].

In practice, however, post-NPM reforms may prove to be a continuation rather than a break with NPM principles. For instance, the introduction of lean in the UK tax agency has been criticized for strengthening the industrial approach to public administration, with focus on efficiency through performance monitoring and a skewed sense of ‘value’ that undermined the public ethos [7]. What ‘value’ means in such reforms, and if the reforms actually change how organizations approach issues of accountability or performance measurements is an empirical question. Hence, there is a need for studies of the practical consequences of reforms, and how they are received and perceived on different levels within public organizations.

In this article, we aim to analyze how the introduction of value-based organizational principles in a sickness insurance agency affects the role of the officials working in the organization. This is done through an empirical investigation of the perceptions of Swedish sickness insurance officials, their office managers, and representatives from the senior management, on the role of sickness insurance officials; how this role has changed after new principles have been introduced; the conditions for officials to fulfil their role in a purposeful way; and how the work is managed and monitored. The data is analyzed through theories on accountability and discretion.

Accountability may be categorized differently depending on which aspects are in focus, e.g., public, administrative, legal, professional or personal accountability [8]. This article focuses on public and professional accountability. Public accountability may be defined as the obligation of a public servant to uphold the public interest, or through a principal–agent framework, in which agents (e.g., public servants) are responsible for acting in the interest of the principal (e.g., the authority), and be answerable to this through rewards or punishments. While public organizations are supposed to work for the benefit of the citizens, the interpretation of public values and what constitutes a public interest may differ over time. Public accountability has under NPM been interpreted through production-oriented managerial models as value at the end of the chain. NPM uses top-down strategies where accountability is measured through constant monitoring of civil servants in order to make them accountable for their actions [9]. Moving towards value-based management could imply a decreased focus on such top-down approaches to accountability, by focusing more on trusting officials’ professionalism; hence, post-NPM reforms will to a larger extent emphasize professional discretion as a key value. Professional accountability refers to the role of the public official and their use of discretion in making decisions, where specific issues are maintaining equity and fairness, while being concerned with the law and procedures [8].

It is not possible to imagine public administration work without professional discretion; it is necessarily embedded in any rule structure, and hence a natural part of officials’ work which they are forced to use [9, 10]. Discretion may be considered as a value in itself, and public encounters considered as positive and necessary for attaining public accountability [11]. There are different elements of discretion [12], defined as rule discretion (limited by legal, fiscal or organizational constraints), value discretion (determined by notions of fairness or codes of conduct), and task discretion (the ability to carry out prescribed tasks). Discretion has often been depicted as a managerial problem, where research has focused on whether it is effective and desirable, and whether government agencies fulfil their goals better through top-down control or through trusting officials to exercise their discretion to deal with complex problems [11]. Increases in rules and accountability may decrease the rule discretion of public servants, although they may still have some discretion in cases where rules are not operable, and task discretion may be high for complex tasks where there are no clear procedures [12]. It has also been argued that discretion always needs to be analyzed in relation to the type and structure of the organizational context, where discretion is influenced both by the level of managerialism and the level of formalization within the organization [13]. Further, discretion is related to the complex networks between professionals and organizations [9, 11] and how organizations are governed. Governing takes place in several layers of an organization [14], where some facets (mainly the productive components) tend to remain more stable while others are aiming to shield the core production from environmental influences in order not to disturb its operations, and as a strategy to reduce uncertainty. On a production level, governing may hence be much focused on managing day-to-day challenges in accordance with established routines and procedures, while governing on a top managerial level may be focusing more on buffering and adapting current managerial and organizational trends external to the organization. This implies that organizations are both open and closed systems at the same time; or, in other words, simultaneously moving targets and relatively stable entities with specific modes of operation. The implementation of new organizational structures or changes in professional roles hence needs to be analyzed on several organizational levels, where changes orchestrated from the top managerial level, from a perspective of a layered organization, may paradoxically contribute to stability and prevent change [15] through discrepancies between hierarchical levels in how reforms are interpreted and carried out (or not carried out). In this article, the notion of layered organizations is used to analyze whether or not the introduction of principles that aim to increase the professional discretion actually will influence the discretion in daily work.

### Swedish Sickness Insurance as a Case

The Swedish sickness insurance system is a general social security system available to all people working in Sweden, and offers income protection in cases of work disability due to illness or injury, regardless of cause. Disability policies in most countries have changed over the last decades, from focusing primarily on passive compensation schemes to promoting activation and integration into work [16]. In this respect, the Swedish sickness insurance system is no exception: since the 1990s there has been a strong focus on activation, e.g., through policy pushes toward promoting job mobility, by introducing time limits and different criteria for work ability assessments at different points [17]. As a consequence, much of the work of sickness insurance officials has become focused on performing eligibility assessments in accordance to the pre-defined time limits. There has also been a general strive toward centralization and standardization, e.g., through centralizing regional offices into a state authority, and through introducing insurance medical guidelines for sick-listing. Much of these changes were introduced while principles of NPM were applied, which included a strong focus on results and performance measures [1, 18].

Recently, The Swedish Social Insurance Agency (SSIA)—the authority in charge of administering most state benefits related to sickness, parental leave, housing allowances, etc.—introduced value-based principles with the ambition to develop the role of the sickness insurance official toward a more holistically oriented case manager. The vision presented was ‘a society where people feel secure if life takes a new turn’, which emphasized the role of the SSIA in offering social insurance services in a timely and reliable way. The SSIA has also promoted certain ‘customer promises’: to be more human, secure and simple, where these promises were developed in dialogue with the employees of the SSIA. To promote a new value-driven agency, the SSIA initiated a set of educational interventions and organizational changes: all officials were given a course in Motivational Interviewing [19], and the organization of the agency was changed into a structure based on their clients’ ‘life situations’ (e.g., being temporary work disabled, or living with a functional disability). A new managerial philosophy was introduced based on ‘trust, respect and compassion’ and a holistic perspective based on creating value for customers. For employees, this also meant the introduction of lean tools [20], and organization of officials into self-governing teams. Examples of lean tools used were value-flow analyses, mapping and simplifying processes, and visualization of results on whiteboards. These changes were introduced primarily as a response to a period of negative media attention and declining trust in the agency, reported in yearly surveys where citizens rate their perceptions of various state agencies. Hence, the changes were driven by internal development projects to improve public legitimacy, rather than by changes in regulation. Similar changes have later been promoted also through government initiatives, with the purpose to promote trustful and quality-oriented management systems.

The SSIA has around 13,500 employees in offices across the country, of which approximately 3500 officials are working with the administration of the sickness insurance system. The system comprises benefits to people on sick leave, where the officials have responsibility for specific cases from the onset of sickness absence and onwards. Insurance officials are to administer pay-outs of benefits, but also to coordinate the rehabilitation process, involving contacts with stakeholders such as healthcare and employers. Officials have mixed educational backgrounds, in recent decades exclusively with university diplomas. When newly recruited, officials receive internal training in regulations and other competences considered necessary to manage the professional role.

Previous research on the role of sickness insurance officials has shown how this group of professionals are generally client-oriented, rather than adherent to regulations [21], especially those with longer work experience. In the last decade, however, the SSIA has had a relatively high employee turnover, combined with increased old age retirement [22], which leads to an increasing number of officials with less work experience. There has also been much pressure on the SSIA during the last decade to lower the number of people on sick leave. This, in combination with the introduction of new regulations, may imply an increasing rule-orientation among insurance officials. Combined with the introduction of new managerial principles, there are thus many demands on sickness insurance officials to balance production requirements (i.e., handling enough cases) and securing a purposeful coordination of individuals’ rehabilitation processes.

## Methods

The data material for this study was originally collected through a project commissioned to study the implementation of Motivational Interviewing into the SSIA, which has been reported in a separate article [23]. In the current article, this material is used to analyze the broader change in managerial principles of which this implementation was a part. The data consists of 57 interviews with employees in different positions within the SSIA, comprising 24 sickness insurance officials in four different offices in the west, east, north and south parts of Sweden; 20 office managers; four regional coordinators; and nine senior management representatives (involving people with strategic functions in the SSIA headquarters, such as analysts and a national insurance coordinator). An overview of the material is presented in Table 1.

**Table 1:** Overview of the material

Office	Office managers	Coordinators	Officials	Senior management
West	1	1	6	
East	2	1	5	
North	1	1	7	
South	2	1	6	
Other	14			9
Total	20	4	24	9

Insurance officials had a variety of educational backgrounds, including social work, political science, human resource management, economics, social sciences, behavioral sciences and nursing. Officials had previous professional experience from different sectors, such as education, private insurance companies, and healthcare. Some officials had worked in the agency for many years and lacked university education. Officials are generally not medically trained, but may consult specialists in insurance medicine when needed in their case work.

### Data Collection

The data collection was carried out between May and September 2013. Four offices were chosen in dialogue with contact persons at the SSIA, where the purpose was to choose offices of average size in middle-sized cities in different regions (the north, south, west and east parts of Sweden). All interviews were semi-structured using an interview guide, covering questions about perceptions of the role of the sickness insurance official, how this has changed over time, and about the implementation and utilization of new tools, such as Motivational Interviewing, lean and teams. Interviews were between 45 and 60 min and were transcribed verbatim. All interviews were carried out in the respondents’ workplaces, apart from three office managers and one coordinator, who were interviewed over the phone.

### Analysis

The analysis was performed in two steps. The first step involved sorting the material according to the principles of a qualitative content analysis [24], where an inductive approach was used. The authors first read through the transcribed interviews repeatedly, to obtain a comprehensive view of the content. Comments and notes were made from first impression, which became the initial coding. Parts of the text that seemed to intercept key thoughts or concepts based on the aim of the study were marked in different colors. The colored parts were then read more systematically with the purpose of organizing the data into categories. Quotes from colored parts of the text were inserted in a separate table. To reduce the text, quotes were condensed into codes describing the data categories. This was made systematically with the first fifteen interviews where officials, coordinators and office managers from the different offices were represented. The remaining interviews were then analyzed in order to confirm the categories, where any opposing data were highlighted. The initial categorization was discussed repeatedly among the authors and continuously during the emerging analysis. Senior management representatives were analyzed separately, where focus was on the more general development of the SSIA over time. Categories identified in this step were (1) the general development of the SSIA; (2) the past and current role of the sickness insurance official; and (3) conditions for managing the current role.

In the second step, a theoretical analysis of the material was carried out. The categories identified in the first step were here related to theories that corresponded to the topics in the material, where issues of accountability and discretion were identified as central for how the respondents described the organizational reforms and the changes in the role of the insurance official. In the analysis, the theories informed an organization of the material into two themes: (1) re-interpretation of professional roles; and (2) managing the implementation of new principles.

### Ethical Considerations

All participants were informed about the purpose of the study and that they could withdraw their participation at any time. The project was approved by the regional ethics board in Linköping (dnr 2013/83-31).

## Results

In this section, the results are presented in two broad themes: the first referring to how the changes in the SSIA has influenced a re-interpretation of the professional role of the insurance official, and the second how the implementation of new organizational principles are managed.

### Re-interpretation of Professional Roles

Within the SSIA, the introduction of value-based principles was a reaction to declining public trust in the agency, following a period of increased restrictions in the sickness insurance system. In the interviews with senior management representatives, the respondents make a general description of the development of the SSIA over the last decade, where a broad image emerges of the SSIA having been too rigorous in their focus on assessing eligibility for sickness benefits, and thereby neglecting their responsibility for coordinating rehabilitation processes. Here, public accountability is raised as a central concern, and is described as a driver of the organizational changes. The centralization of the SSIA from several regional to one national authority is mentioned as an explaining factor for the previously strong focus on standardization. After this re-organization has settled, the pendulum now shifts toward a more client-centered way of working in order to meet expectations from the public. A senior management representative notes how today’s role demands flexibility on the side of the official in coordinating stakeholders and adapting their actions to different circumstances, which calls for more discretion.

The discretion was probably greater 10 years ago; the process wasn’t as structured, not as detailed as it is now. So, I definitely think officials perceive having less discretion. And I think that’s counter-productive, since there’s many stakeholders involved, and much variety in sick leave cases. There’s different solutions needed, and officials need to act with flexibility and adapt their actions to the situation (National insurance coordinator).

Managers also emphasize how the public lacks knowledge about the current insurance regulations; there are unrealistic expectations of what the SSIA can offer their clients, where such expectations are reflections of the more generous system of the past. This understanding of public accountability as trust in the agency has introduced new interpretations of professional accountability, where officials are expected to focus more on how they are meeting clients and their pedagogical responsibility in describing the current system in order to manage expectations.

Officials also describe how their role has changed. Previously, their work was detail-oriented and all cases should be handled in a standardized way. In the present role, the work is described as gentler and broader with a holistic perspective of the client. Further, the role is now concerned with the entire process of a client, compared to how officials previously could be responsible for only parts of that process. However, officials with a longer experience note how the current role in a historical perspective is much more controlled with less discretion for officials to decide upon their work. Today’s role is more structured, and the performance of the officials is measured in greater detail, which affects what is prioritized.

It’s much more regulated today, what we are supposed to be doing. There are a number of measurements of our work and more goals, so it’s more structured today. 10 years ago, you were expected to work toward a broader goal, such as bringing people back to work, or shorten the sick leave spells, or retire people, those kinds of overarching goals. There weren’t these goals with numbers attached to them, such as managing an application within a certain number of days, or making a specific assessment within 180 days. It changes the work entirely (Insurance official 4, office 2).

That managers and officials describe a shift from administration to case management may thus be seen, at least partly, as a shift back to how the role was perceived before the SSIA was centralized, and before the regulations became stricter. The values introduced into the SSIA in recent years are focusing on service-oriented, efficient and fair management of cases. Officials also emphasize working with coordination activities in order to promote stakeholder collaboration, and to promote the clients’ own responsibility and involvement in their rehabilitation process. This is seen as related to changes in regulations where individual responsibility is more clearly emphasized.

To have this coordinating role is important, that people on sick leave can feel that they can talk to us. A plan where this person is helped in taking a new step, so that it may be more sustainable (Insurance official 1, office 1).

Officials describe their role as divided, as their responsibilities tends to move in two different directions, with focus on administration on the one hand, and coordination on the other. Managers clearly point out that the officials’ role should not therapeutic, which may be interpreted as a resistance to officials taking on too much responsibility for their cases.

You should be clear about that we are no therapists. We should listen to what is needed and try to arrange so that others do what they are supposed to. So, we shouldn’t be too caring, it isn’t our job. If you are, I think you will have problems managing it (Office manager 1, office 1).

The respondents identify several skills required for officials. Both office managers and officials describe a complex role where knowledge about the insurance and legislation is of central importance, as is the ability to deal with people. Further, the respondents mention the need of both experience-based knowledge and formal higher education.

I’m not sure we have been up to date in this development, in supplying the officials with knowledge and competence. You have to be holistic and see the context to come up with good solutions, and it’s around that we have started to think about how to best professionalize our officials for this. Because this is a tough task, where many stakeholders are involved, and we are supposed to coordinate it (National insurance coordinator).

Several managers emphasize the importance of higher education for officials. Still, young academics have less life experience compared to older officials, which may have an impact on the professional ability to address the client’s situation. A few managers also stress that it may be easier for the organization to form employees with less education. Hence, managers express an ambivalent stance toward the required skills of officials, where they simultaneously need to be educated enough to practice professional discretion, and enough obedient to comply with organizational procedures.

Discretion is described as embedded in a context of routines and procedures that are quite restrictive. Officials have discretion to decide over their daily agenda and planning (i.e., task discretion), although within the limits of a structured routine anchored in the time limits in the sickness insurance regulations (rule discretion). Hence, the officials view their current role as both more controlled than previously (due to regulatory changes and a recent history of micromanagement), and as involving more professional discretion (due to the new focus on case management). The SSIA of the past is described as having broader objectives and less detailed procedures and routines, and the SSIA under NPM as heavily structured through performance measures and micro-management. The accounts from the officials suggest that the current SSIA appears to be a mix of the two. Here, the professional accountability is interpreted as dependent on the professional skills of the official in meeting clients and managing cases, linked to an interpretation of public accountability into values related to client-orientation. The current approach to professional accountability is however still influenced by NPM, as the administrative routines are abundant and performance is heavily monitored.

### Managing the Implementation of New Principles

Both office managers and officials with longer work experience describe the SSIA as an organization where changes in what is prioritized comes and goes with a certain regularity, and that they are therefore accustomed to reforms. Most of the interviewed officials had however been employed for a shorter time, and did not share these experiences of previous changes. Notably, most interviewed officials had been employed after the SSIA was centralized and stricter eligibility criteria were introduced into the sickness insurance system. An official who was employed in 2003 explains how the focus has shifted over the years:

When I started, we were just leaving one way of managing sick leave cases, where we were, what can I say… more generous. […] Then we entered a period with rising sick leave numbers, where we had increasing caseloads and it became a political issue that sick leave rates had to come down. It changed the atmosphere completely, and case management changed. We were told to end sick leave cases. And that didn’t turn out good, I don’t think many officials enjoyed working then, and we had to take a lot of frustration from people on sick leave. So that was a bit rougher. But now it has changed again (Insurance official 3, office 2).

While the changes toward client-orientation are broadly welcomed, it can be noted how the organizational culture changes slowly and that previous principles based on NPM and traditional public administration co-exist with new initiatives. Some officials express a certain weariness with the constant reforms and indicate that they usually work ‘as usual’ anyway. Leadership emerges as an important aspect of the change process, where the office managers carry much of the organizational culture and values in their managerial style, which still appears to be much influenced by NPM. One manager express how there needs to be a balance between change and stability:

The work never stands still, but at the same time I hope we have employees who likes that there’s always a development, a change. But it cannot be too much of those things; I can worry about that (Office manager 10).

Senior management representatives express how the new principles should lead to managers shifting from the previous system of micromanaging to focusing on coaching and emphasizing professionalism, e.g., through delegating responsibilities to teams of officials.

To show that you have faith in the employees and the work they are doing, and supply competence in the right direction, so to speak. To manage through knowledge and competence instead of numbers and statistics. I am personally convinced that this is a recipe for success (Competence manager).

This is not mirrored by the officials, who express how their performance is still very much measured in terms of quantitative production quotas. Hence, the NPM principles still linger in the workplace culture, and, according to the officials, the rhetoric of increased discretion is challenged by the plethora of routines. The public accountability of officials is, as a consequence, still perceived as being much determined by keeping to procedure, rather than by the quality of encounters with the clients. Performance measures do not appear to have changed when the new principles were introduced, and there are no strategies for determining the quality of meetings, or whether officials are using the proposed client-oriented methods in order to promote RTW (e.g., Motivational Interviewing).

Management representatives describe the recent changes as a coherent development, where the introduction of lean, teams and Motivational Interviewing are seen as parts of a larger strategy. Among officials, on the other hand, the various changes are generally seen as separate from each other, where it is uncommon to link these changes to a broad perspective of how the SSIA is moving toward a new direction. It is illustrative how the changes toward team and lean organization by some officials is considered to be examples of focusing on effectiveness rather than quality:

Motivational Interviewing is pretty far from the numbers, from the results and the graphs and everything. It becomes more of a quality issue, which is often lost when we speak about this team and lean stuff; that’s more focused on the work, meaning results and effectiveness, in a way (Insurance official 4, office 2).

This indicates different interpretations in different layers of the organization, where the broad picture emphasized by management is not as common among the officials. Although officials do not connect the specific reforms to one another or to an overall strategy, they do however describe a general development of their role as moving from being an administrator focusing on assessing eligibility, to becoming a case manager, implying that they have perceived the broad orientation of the organizational changes.

Officials appear to struggle to balance the production demands of the line organization with the more client-oriented values that are currently promoted by the senior management. They mention how the constant stream of new initiatives from the management tend to increase their workload, resulting in increasingly poor working conditions. The heavy workload makes the work challenging, especially as the role becomes increasingly complex. The changes toward a more client-centered approach with more focus on coordination result in greater demands on officials to participate in different meetings, since other stakeholders are requesting their presence. Officials describe how the high workload and the need to attend meetings inhibit their ability to fulfil their role in relation to the client, and that they have to prioritize the most urgent issues (most often securing pay-outs of sickness benefits). Officials also express difficulties related to the coordinating function, where they do not have control over other stakeholders’ activities, e.g., waiting lists in healthcare. Further, different views on the situation of the client among stakeholders can obstruct the rehabilitation process, as can other stakeholders’ lack of understanding for the role and responsibilities of the SSIA.

When it comes to employers [of sick-listed clients], I can’t do very much since they have their [concerns], you have to accept that. And healthcare has their waiting lists, and that I cannot influence at all (Insurance official 5, office 1).

It is likely that the more complex tasks, such as coordinating rehabilitation processes, managing stakeholder interactions and promoting RTW, are those requiring the most professional discretion, since routines and regulations are less detailed in this area. When working conditions force officials to focus on core tasks, the room for discretion diminishes, as do the room for reflection and the possibility for officials to engage in continuous improvements. For instance, the introduction of teams is mentioned as positive, although the officials in the present study primarily used the teams for scheduling, and not for peer consultation or developmental work.

## Discussion

Respondents in the study describe a pendulum between standardization and client-orientation, where officials were given an increasing responsibility for managing their daily work, while still needing to comply with a complex set of regulations and routines. The recent shift of the pendulum is toward more task and value discretion, albeit within the limits of a strict legislative framework, which limits the rule discretion. Further, the task discretion is limited by detailed administrative routines. The officials in this study welcome increasing discretion since it facilitates the complex tasks of managing stakeholder coordination and promotion of RTW. On the other hand, they are struggling to manage the different concurrent management principles within the organization. While lean and team organization are being put forward on an organizational level, performance measures based on NPM and traditional bureaucratic principles are still prioritized in the daily work and in the feedback given by managers.

The introduction of new principles was due to a crisis in public trust in the system. Given that trust is mutual, this can be related to studies of how systems differ in how well they trust their clients, where social-democratic welfare regimes generally are more trusting [6]. It may be argued that the activation policies and the NPM principles has caused the sickness insurance system to deviate from the elements that generated high public trust. As reflected in previous research, NPM has been used as a tool to introduce the activation paradigm into disability policies, where caseworkers have internalized this into a belief system where a good caseworker is a person who has understood the notion of activation, and hence the importance of being more strict in relation to clients [25]. It may be argued that the NPM principles are much in line with such policies, and that these values are still strong within the organization.

Implementation of organizational reforms are complex, and often lead to only parts of the intended results. Since public organizations are embedded in administrative traditions forming an institutional path dependency, new ways of organizing and managing officials’ work may be difficult to implement [26, 27]. Further, the notion of “value-based” principles is slippery, and the promoted values may be transformed during the implementation process. For instance, studies has pointed out how the adaptation of lean is often narrow when implemented, and often limited to certain tools [3], most likely those that are in line with the current principles and procedures within an organization and therefore are considered easier to implement. In this case, the use of lean tools to promote client-oriented values is complicated by the authoritative context of a state agency, and the NPM principles and performance measures. Holmgren et al. [28] argue that there are three parallel management principles in today’s SSIA: the traditional public administration model with focus on bureaucracy and regulations; balanced scorecard management inspired by NPM with focus on results through detailed measures of performance; and lean, focusing on value for customers and efficient processes. These principles may complement each other, but may also come into conflict. The challenge of introducing lean in public organizations with strong cultures and structures has been recognized in previous literature [2], either since the change is in conflict with professional values, or because they are implemented top-down which causes frontline staff to focus more on internal measures and targets than on the end-users. In this case, one ambition of introducing new principles was that officials, through using lean, should be contributing to continuous improvements, which in a context of an authority is complicated by the amount of legislation that governs the work of the employees, and where the organizational culture is hierarchical and rule-oriented.

A previous study of the recent reforms in the SSIA has pointed out that the number of rules have not decreased, which makes the application of professional discretion through team work complicated and frustrating for officials, where rule- and result-orientation is still heavily prioritized by the management [29]. The recent reforms may hence be seen as supplements to the existing NPM paradigm, rather than as a distinctive break with it. Officials’ discretion is limited to small details, while the overall work routines are much controlled: they are struggling with their professional discretion in relation to the legal demands, the detailed work routines and the recent history of micromanagement. The new value-based principles also place large demands on the front-line management, especially in how to combine safeguarding fundamental adherence to insurance regulations with supporting professional discretion, i.e., keeping a balance between discretion and governance. In turn, officials balance not only consideration toward clients and obedience to legislation, but also loyalty to their superiors [30], and hereby to the organizational culture that managers project through their management style.

This discrepancy between the rhetoric of the senior management and the practice of office managers and officials may be seen through the lens of a layered organization [14], in which the senior management adapts to new values in order to meet expectations from the public, while other layers (office managers) are still under the influence of how the organization measures its results. This creates a de-coupling of the managerial (and externally-oriented) rhetoric of the organization from the actual work performed by officials, where office managers’ NPM-based strategies effectively shield off the influence of the rhetoric from how the work is monitored and rewarded.

The reforms introduced to promote professional discretion may be seen as an initiative to prevent negative effects of detailed top-down management, while the complications surrounding its implementation points to challenges in promoting discretion in a state authority governed by strict legislation. The material displays an interesting combination of bottom-up rhetoric (teams being drivers of innovation and flexible case management) and top-down management strategies (micromanagement, a strong focus on organizational order and routines, and detailed regulations). In their promotion of professional discretion, the SSIA has largely used top-down strategies where the officials, used as they are in following orders, have carried out the required changes, e.g., establishing teams. The actual changes, however, are limited in scope due to the high workload of officials, which is largely managed by falling back into the routines established in the previous NPM paradigm (also illustrated by the largely failed implementation of Motivational Interviewing, as reported elsewhere [23]). While top-down and routine-based strategies may be effective for repressing doubt and promoting standardized work routines, they may be destructive for employees’ creativity and use of intellectual resources [31]. This, in turn, may be related to the conditions for officials to perform their work in an ethically sound way (value discretion), where the application of regulations needs to be balanced with attention to characteristics of the individual client. It may be argued that managing that balance requires an organization and a management that does not suppress officials’ intellectual abilities or room to question the application of rules, especially in situations where routinized actions may have serious consequences.

The purpose of the reforms was to improve client satisfaction and public legitimacy through more client-oriented procedures. A possible implication for clients of introducing value-based management principles is, therefore, that they receive services that display more respect for the details of their individual case and hence get more adequate support in managing their situation. Although this study did not study clients’ perceptions, we can conclude from the data that the reforms did not have the desired effect on procedures, since previous organizational principles prevailed and complicated the introduction of new approaches. Finally, it should be mentioned that yet newer reforms have taken place since the data in this article was collected, which again shifts the balance between discretion and governance, towards the latter. At the time of writing, the SSIA has had a change of Director-General, which tends to lead to new reforms, and Sweden has had a change of government, which tends to lead to new policies. The influence of leaders and government policies on the practices in public agencies, and as a consequence on the services provided to clients, is outside the scope of this article, but may be an important topic for future research in this field.

### Methodological Considerations

This is a qualitative study, where the perceptions and attitudes reported are to be seen as representative of the informants, and not necessarily of the SSIA as a whole. The results are however much in line with previous studies of the SSIA, both qualitative and quantitative, which strengthen the trustworthiness of the results. Since this is a single-case study, the results are limited to a Swedish sickness insurance context; the results may however be transferred to studies of public organizations in other contexts, where similar developments in management and organizational principles are described.

## Conclusions

This study illustrates how re-interpretation of public values may lead to a change in how public and professional accountability is defined, where the focus shifted from routines and performance measurements toward professional discretion and the quality of encounters. This development is in line with current evidence on work disability prevention and promotion of RTW, where trustful cooperation structures between the central stakeholders is commonly argued for. However, the results also show how these cultural changes were interpreted differently across different layers of the organization, which illustrates the complexities of introducing changes. The NPM discourse is strong within many public organizations where accountability is commonly managed through top-down performance measures; the results point to the lingering influence of NPM which makes it challenging to promote discretion and client-centered principles. It is therefore important for public organizations to reconcile new organizational principles with the current organizational culture and how this is manifested through managerial styles, which may be resistant to change.
